# Correction: Rondanelli et al. Effectiveness of a Novel Food Composed of Leucine, Omega-3 Fatty Acids and Probiotic *Lactobacillus paracasei* PS23 for the Treatment of Sarcopenia in Elderly Subjects: A 2-Month Randomized Double-Blind Placebo-Controlled Trial. *Nutrients* 2022, *14*, 4566

**DOI:** 10.3390/nu17213424

**Published:** 2025-10-31

**Authors:** Mariangela Rondanelli, Clara Gasparri, Gaetan Claude Barrile, Santina Battaglia, Alessandro Cavioni, Riccardo Giusti, Francesca Mansueto, Alessia Moroni, Fabrizio Nannipieri, Zaira Patelli, Claudia Razza, Alice Tartara, Simone Perna

**Affiliations:** 1 IRCCS Mondino Foundation, 27100 Pavia, Italy; mariangela.rondanelli@unipv.it; 2 Unit of Human and Clinical Nutrition, Department of Public Health, Experimental and Forensic Medicine, University of Pavia, 27100 Pavia, Italy; 3 Endocrinology and Nutrition Unit, Azienda di Servizi alla Persona “Istituto Santa Margherita”, University of Pavia, 27100 Pavia, Italy; gaetanclaude.barrile01@universitadipavia.it (G.C.B.); alessandro.cavioni01@universitadipavia.it (A.C.); francesca.mansueto01@universitadipavia.it (F.M.); alessia.moroni02@universitadipavia.it (A.M.); zaira.patelli01@universitadipavia.it (Z.P.); claudia.razza01@universitadipavia.it (C.R.); alice.tartara01@universitadipavia.it (A.T.); 4 Medical Research Abiogen Pharma, 56121 Pisa, Italy; santina.battaglia@abiogen.it (S.B.); riccardo.giusti@abiogen.it (R.G.); fabrizio.nannipieri@abiogen.it (F.N.); 5 Department of Biology, College of Science, University of Bahrain, Sakhir Campus, Zallaq P.O. Box 32038, Bahrain; simoneperna@hotmail.it

Error in Table

In the original publication [1], there was a mistake in Table 1 as published. The values of ALM are incorrect. The values needed to be changed, expressing them in kg and not g. So, the correct values are as follows: 15.646 ± 3.666; 13.935 ± 2.020; and 14.773 ± 3.03. The corrected Table 1 appears below. 

In the original publication, there was also a mistake in Table 2 as published. The symbol “>“ is in the wrong direction. The symbol needed to be in the opposite direction (i.e., changed from “>“ to “<“). Moreover, the unit of measurement of ALM is not correct; the correct unit of measurement of ALM should be “kg” instead of “g”. The values reported in the table are correct. The corrected Table 2 appears below. 

The authors state that the scientific conclusions are unaffected. This correction was approved by the Academic Editor. The original publication has also been updated.

## Figures and Tables

**Table 1:** The baseline clinical characteristics of patients between the intervention and placebo groups.

Variable	Intervention Group (*n* = 22)	Placebo Group (*n* = 28)	Total (*n* = 50)	*p*-Value
Age (years)	78.84 ± 5.80	80.50 ± 3.74	79.71 ± 4.84	0.231
Weight (kg)	57.53 ± 11.41	52.32 ± 8.33	54.77 ± 10.14	0.072
BMI (kg/m^2^)	22.63 ± 2.74	21.96 ± 1.34	22.27 ± 2.12	0.271
ALM (kg)	15.646 ± 3.666	13.935 ± 2.020	14.773 ± 3.035	0.052
ALM/h2	6.20 ± 1.23	5.80 ± 0.83	6.00 ± 1.04	0.270
SMI	27.40 ± 4.98	26.61 ± 4.18	26.99 ± 4.60	0.521
FM (g)	18,701.52 ± 4953.04	16,474.92 ± 4516.35	17,520.06 ± 4809.19	0.106
VAT (g)	898.48 ± 648.860	698.04 ± 238.10	792.12 ± 482.40	0.149
WC (cm)	81.22 ± 6.95	79.46 ± 8.14	80.29 ± 7.58	0.424
DBP (mm Hg)	77.30 ± 4.19	78.65 ± 5.08	78.02 ± 4.68	0.319
SBP (mm Hg)	126.96 ± 5.59	125.58 ± 5.89	126.22 ± 5.73	0.406
Tinetti (score)	17.83 ± 4.01	16.88 ± 3.70	17.33 ± 3.84	0.397
SPPB total (score)	4.22 ± 1.45	4.38 ± 0.64	4.31 ± 1.08	0.595
Handgrip (kg)	18.74 ± 4.33	17.64 ± 4.68	18.06 ± 4.52	0.328
Barthel (score)	60.43 ± 18.64	55.58 ± 19.30	57.86 ± 18.96	0.376
ADL (score)	3.22 ± 0.85	3.19 ± 1.50	3.20 ± 1.22	0.944
Physical SF-12 (score)	33.80 ± 6.83	37.29 ± 11.82	35.65 ± 9.86	0.220
Mental SF-12 (score)	40.78 ± 8.73	40.89 ± 12.40	40.83 ± 10.56	0.973
GDS (score)	14.13 ± 1.98	13.88 ± 1.77	14.00 ± 1.86	0.649
Creatinine (mg/dL)	0.86 ± 0.13	0.79 ± 0.21	0.82 ± 0.18	0.181
AST (IU/L)	19.26 ± 8.86	18.04 ± 8.81	18.61 ± 8.77	0.631
ALT (IU/L)	16.17 ± 7.99	16.38 ± 10.87	16.29 ± 9.53	0.939
GGT (IU/L)	15.90 ± 3.15	16.37 ± 2.39	16.15 ± 2.75	0.560
FBG (mg/dL)	86.09 ± 11.16	88.92 ± 9.43	87.59 ± 10.27	0.340
CRP (mg/dL)	1.20 ± 1.45	0.94 ± 0.59	1.06 ± 1.08	0.392
Leucine	105.30 ± 18.33	99.95 ± 24.19	102.46 ± 21.59	0.393
Isoleucine	88.25 ± 24.09	88.63 ± 25.47	88.45 ± 24.58	0.958
Valine	133.30 ± 36.23	143.98 ± 41.39	138.97 ± 39.02	0.344
Total AA	326.85 ± 38.43	332.56 ± 57.64	329.88 ± 49.15	0.689

Abbreviations: AA, amino acid; ADL, activity daily living; ALM, appendicular lean mass; ALT, alanine aminotransferase; AST, aspartate aminotransferase; BMI, body mass index; CRP, C-reactive protein; DBP, diastolic blood pressure; FBG, fasting blood glucose; GDS, geriatric depression scale; GGT, gamma-glutamyl transferase; SPPB, short physical performance battery; SBP, systolic blood pressure; SF-12, 12-item short-form health survey; VAT, visceral adipose tissue; WC, waist circumference.

**Table 2:** Primary and secondary endpoints.

Variable	Intervention Group (N = 22) Mean Change from Baseline (95% CI)	Placebo Group (N = 28) Mean Change from Baseline (95% CI)	Difference between Groups (95% CI)	*p*-Value between Group
Weight (kg)	1.378 (0.813, −1.943)	−1.442 (−1.972, −0.912) *	2.820 (2.028, 3.611)	<0.001
BMI (kg/m^2^)	0.539 (0.308, 0.769) *	−0.600 (−0.816, −0.384) *	1.139 (0.816, 1.462)	<0.001
ALM (kg)	0.350 (−0.607, 1.307)	−1.268 (−2205.44, −332.26) *	1.618 (−255.75, 2982.00)	0.245
ALM/h2	0.516 (−0.114; 1.146)	−0.505 (−1.103; 0.097)	1.021 (−1.910; −0.132)	<0.05
SMI (%)	0.282 (−1.098; 1.662)	−0.950 (−2.269; 0.368)	1.232 (−0.719; 3.170)	0.208
FM (g)	700.522 (360.688, 1040.356) *	−646.962 (−965.724, −328.200) *	1347.484 (871.386, 1823.582)	<0.001
VAT (g)	−113.171 (−188.061, −38.280) *	9.151 (−61.096, 79.398)	−122.321 (−227.241, −17.402)	<0.001
WC (cm)	0.441 (−0.005, 0.887)	−0.582 (−1.000, −0.164) *	1.023 (0.399, 1.648)	<0.001
DBP (mm Hg)	0.214 (−1.364; 1.764)	−0.574 (−2.055; 0.907)	0.788 (−1.423; 3.000)	0.477
SBP (mm Hg)	−0.332 (−2.004, 1.341)	−0.399 (−1.968, 1.170)	0.067 (−2.276, 2.411)	0.954
Tinetti (score)	1.940 (0.989, 2.891) *	−0.447 (−1.339, 0.446)	2.386 (1.054, 3.719)	<0.001
SPPB total (score)	2.667 (2.108, 3.226) *	0.448 (−0.076, 0.973)	2.219 (1.436, 3.002)	<0.001
Handgrip (kg)	3.332 (2.400, 4.264) *	−0.755 (−1.629, 0.119)	4.087 (2.781, 5.392)	<0.001
Barthel (score)	4.222 (0.480, 7.964) *	0.111 (−3.399, 3.621)	4.111 (−1.132, 9.353)	0.121
ADL (score)	0.506 (0.127, 0.885) *	−0.101 (−0.457, 0.254)	0.607 (0.076, 1.138)	<0.001
Physical SF-12 (score)	2.747 (−0.472, 5.966)	3.519 (0.499, 6.538) *	−0.772 (−5.281, 3.738)	0.732
Mental SF-12 (score)	1.238 (−0.631, 3.107)	3.789 (2.036, 5.543) *	−2.551 (−5.170, 0.067)	0.056
GDS (score)	−0.262 (−0.631, 0.106)	0.155 (−0.190, 0.501)	−0.418 (−0.934, 0.099)	0.110
Creatinine (mg/dL)	−0.025 (−0.078, 0.027)	0.043 (−0.007, 0.092)	−0.068 (−0.141, 0.005)	0.069
AST (IU/L)	0.421 (−2.311, 3.154)	0.435 (−2.128, 2.998)	−0.013 (−3.841, 3.815)	0.994
ALT (IU/L)	3.150 (0.042, 6.258) *	0.059 (−2.856, 2.974)	3.091 (−1.263, 7.445)	0.160
GGT (IU/L)	0.235 (−0.236, 0.705)	0.069 (−0.372, 0.511)	0.165 (−0.494, 0.824)	0.617
FBG (mg/dL)	−0.904 (−5.580, 3.773)	−2.470 (−6.856, 1.917)	1.566 (−4.986, 8.118)	0.633
CRP (mg/dL)	−0.690 (−1.094, −0.286) *	0.266 (−0.113, 0.645)	−0.956 (−1.522, −0.390)	<0.001
Leucine	17.832 (11.653, 24.010) *	4.814 (−0.981, 10.610)	13.017 (4.361, 21.674)	<0.001
Isoleucine	17.855 (13.369, 22.341) *	−2.304 (−6.512, 1.903)	20.159 (13.875, 26.444)	<0.001
Valine	24.020 (16.177, 31.863) *	−3.116 (−10.472, 4.241)	27.135 (16.148, 38.123)	<0.001
Total AA	59.706 (47.960, 71.452) *	−0.606 (−11.623, 10.412)	60.312 (43.856, 76.768)	<0.001

* *p* value set up at <0.001 Abbreviations: AA, amino acid; ADL, activity daily living; ALM, appendicular lean mass; ALT, alanine aminotransferase; AST, aspartate aminotransferase; BMI, body mass index; CI, confidence interval; CRP, C-reactive protein; DBP, diastolic blood pressure; FBG, fasting blood glucose; GDS, geriatric depression scale; GGT, gamma-glutamyl transferase; SPPB, short physical performance battery; SBP, systolic blood pressure; SF-12, 12-item short-form health survey; VAT, visceral adipose tissue; WC, waist circumference.
